# Randomized Single-Blinded Non-inferiority Trial Of 7 mg/kg Pentamidine Isethionate Versus 4 mg/kg Pentamidine Isethionate for Cutaneous Leishmaniaisis in Suriname

**DOI:** 10.1371/journal.pntd.0003592

**Published:** 2015-03-20

**Authors:** Ricardo V. P. F. Hu, Masja Straetemans, Alida D. Kent, Leslie O. A. Sabajo, Henry J. C. de Vries, Rudy F. M. Lai A Fat

**Affiliations:** 1 Dermatology Service, Ministry of Health, Paramaribo, Suriname; 2 Department of Biomedical Research, Royal Tropical Institute, KIT, Amsterdam, The Netherlands; 3 Department of Parasitology, Anton de Kom University of Suriname, Paramaribo, Suriname; 4 Centre for Infections and Immunity Amsterdam (CINIMA), Academic Medical Center, University of Amsterdam, Amsterdam, The Netherlands; 5 Department of Dermatology, Academic Medical Center, University of Amsterdam, Amsterdam, The Netherlands; 6 STI Outpatient Clinic, Cluster Infectious Diseases, Public Health Service Amsterdam, Amsterdam, The Netherlands; 7 Department of Dermatology, Academic Hospital, Paramaribo, Suriname; AP-HP, service de parasitologie-mycologie, FRANCE

## Abstract

**Background:**

Standard treatment of cutaneous leishmaniasis (CL) in Suriname entails three injections of pentamidine isethionate (PI) 4 mg/kg per injection in 7 days (7 day regimen). Compliance to treatment is low and may contribute to increasing therapy failure. A 3 day regimen, including 2 injections of 7 mg/kg in 3 days may increase compliance.

**Methods:**

In a randomized, single-blinded non-inferiority trial conducted in Suriname, 84 CL patients received the 7 day regimen and 79 CL patients received the 3 day regimen. Primary objective was the proportion of patients clinically cured at 6 weeks follow-up. Secondary objectives were clinical cure at 12 weeks follow-up; parasitological cure at 6 and 12 weeks; adverse and drug related toxicity events recorded one week after the end of treatment and health related quality of life. The non-inferiority margin was set at 15%, 1 sided test, α = 0.1.

**Results:**

At 6 weeks follow-up 31 (39%) patients in the 3 day regimen and 41 (49%) patients in the 7 day regimen were clinically cured. Intention to treat (ITT) analyses showed that the difference in proportion clinically cured was -9.6% (90% Confidence Interval (CI): -22.3% to 3.2%). Per protocol (PP) analysis showed that the difference in proportion clinically cured was 0.2% (90% CI: -14.6% to 15.2%). ITT analysis showed that the difference in proportion parasitological cured at 6 weeks was -15.2% (90% CI:-28.0% to -2.5%). PP analyses showed similar results. Non-inferiority could not be concluded for all adverse and toxicological events.

**Conclusion:**

We cannot conclude that the 3 day regimen is non-inferior to the 7 day regimen regarding proportion clinically and parasitological cured. Therefore there is no evidence to change the current standard practice of the 7 day regimen for the treatment of CL in Suriname.

## Author Summary

Cutaneous Leishmaniasis (CL), caused by the single cellular protozoan parasite of the genus *Leishmania*, is treated in Suriname with pentamidine isethionate (PI). Standard treatment consists of 3 intramuscular injections of 4 mg per kg bodyweight given in 7 days. Compliance to treatment is low which may lead to treatment failure. A shorter treatment course of PI, 3 days, may aid in solving the compliance problem. In this study CL patients were included in two study arms, the control arm, PI 4 mg/kg/injection, three injections in 7 days (84 patients) and the intervention arm, PI 7 mg/kg/injection, two injections in 3 days (79 patients). Based on the study results it cannot be concluded that that the 3 day regimen is at least as good (non-inferior) to the 7 day regimen, as far as clinical and parasitological cure, toxic and adverse events. Based on these findings there is no evidence to change the standard treatment of 3 injections of 4 mg/kg PI in Suriname.

## Introduction

With a global yearly incidence around 1.5 million cases, Cutaneous leishmaniasis (CL) is the most common clinical presentation of Leishmania infection.[1] The bite of an infected female sandfly of the genus *Phlebotomus* and *Lutzomyia* transmit the protozoan *Leishmania* parasites.[2]

In Suriname CL is endemic and, mainly caused by *L*. *(V*.) *guyanensis*. Pentamidine isethionate (PI) is the preferred treatment.[3] The standard treatment regime consists of three intramuscular injections of 300 mg PI salt per injection in 7 days. Low treatment compliance is a general and major problem.

Van der Meide et al. found that only 50% of the patients received the complete three injections therapy.[4] Many patients with CL travel from the interior to the capital city Paramaribo for diagnosis, subsequent treatment and then return to the forest with PI for self-administration. Without proper medical supervision incomplete therapy and sub-therapeutic PI blood levels are common, which may contribute to the development of drug-resistant parasites.[5]

A shorter treatment regimen might improve compliance based on our beliefs that noncompliance is caused by the costs involved in seeking help for CL and the consequential income loss. A treatment regime of either one or two intramuscular injections with 7 mg/kg PI salt in 3 days, evaluated in a retrospective French Guyanese study, indicated similar cure rates for the non-severe cases.[6] Lost to follow-up was lower in the one injection regimen (35%) compared to the two injections regimen (46%). Limitations of the study were: lost to follow-up patients were considered cured in the intention to treat analysis, side effects were limited to pain at injection site and neuropathy, and toxicity of PI and laboratory abnormalities were only mentioned briefly.

In previous studies the 7 mg/kg PI salt regimens have never been evaluated as opposed to the 4 mg/kg regimen in controlled trial settings. Therefore, we have conducted a randomized single-blinded non-inferiority trial to assess if a 3 day injection regimen consisting of 7 mg/kg PI is not worse (non-inferior) with respect to clinical and parasitological cure rate, adverse events and health related quality of life (HRQL) compared to the standard 7 day PI regimen for the treatment of CL patients visiting the dermatological outpatient clinic in Paramaribo, Suriname.

## Methods

### Ethics statement

This study was approved by the Medical Ethical Commission of the Ministry of Health Suriname (VG 006-2009). Written informed consent has been obtained from all participants.

We conducted a randomized single-blinded non-inferiority trial from January 3^rd^ 2010—April 30^th^ 2013 at the outpatient clinic of the Dermatology Service in Paramaribo. The study was approved by the ethical review commission of the Suriname Ministry of Health and registered at the Dutch Trial Register (NTR 2076).

Eligible individuals were ≥ 16 years with laboratory confirmed CL (histopathology and/or Giemsa smear of biopsy) who could be contacted by phone. Exclusion criteria were CL patients treated in the previous six months, pregnancy or lactation, unable to attend one of the study visits, medical history of diabetes mellitus, cardiac-, renal- and hepatic disease, abnormal baseline values for amylase, aspartate aminotransferase and alanine aminotransferase, creatinine, glucose, hemoglobin, leucocytes, thrombocytes and patients with known allergy to PI.

After written informed consent was provided, patients were randomized for the intervention regime of two intramuscular injections of 7 mg/kg PI salt on days 1 and 3 (3 day regimen) or the control regime of three intramuscular injections of 4 mg/kg on days 1, 4 and 7 (7 day regimen). Injections were administered by the dermatologist aware of the intervention allocation. Randomization was performed by a computerized balanced block randomization scheme that was stratified on disease severity based upon the presence or absence of clinical loco regional lymphadenitis.

The primary objective was to establish if the 3 day regimen is non-inferior to the 7 day regimen regarding the primary endpoint clinical cure six weeks after end of treatment.

The secondary endpoints were clinical cure at 12 weeks, parasitological cure at 6 and 12 weeks, adverse and drug related toxicity events one week after the end of treatment and HRLQ differences before treatment and at 6 weeks follow-up visit.

Clinical evaluation was conducted at enrollment, during the treatment visits and during the follow-up visits 1, 6 and 12 weeks after treatment. Lesions were measured, recorded and photographed. If more than one lesion existed, the largest lesion was followed up. Lymph tracks and regional lymph node stations were palpated. Questionnaires for HRQL (Skindex-29 [7] and EQ-5D/VAS [8]) and adverse events were recorded. When participants suffered from therapy failure at follow-up visits 6 or 12 weeks, the standard 7 day regimen was additionally offered. Upon inclusion, two skin biopsies of 2 mm were taken of the largest lesion. Parasitological diagnosis was established by microscopic examination of biopsy smears and histopathology. For parasite quantification, Nucleic Acid Amplification based methods were performed on biopsies before treatment and at weeks 6 and 12. [9,10] Laboratory values were evaluated one week after treatment to monitor potential drug related toxicity. Blood pressure was measured before, during- and 30 minutes after each injection.

Clinical cure was defined as complete re-epithelization and absence of inflammatory signs (infiltration, erythema and/or scaling). Therapy failure was observed in case of incomplete re-epithelization and/or inflammatory signs. Two independent blinded dermatologists with at least four years of diagnostic CL experience determined clinical cure (using standardized photographs of lesions). In case of disagreement a third blinded dermatologist passed the final judgment.

Parasitological cure was defined as a log3 decrease in parasite load in the follow up lesion at visits 6 and 12 weeks compared to baseline. The SKINDEX-29 questionnaire was used to measure the HRLQ.[7] With the EQ-5D and EQ-VAS the generic QoL was measured.[8]

With a structured questionnaire, patient reported adverse events were identified throughout treatment and follow-up. Drug related toxicity; drop in diastolic blood pressure > 10mm Hg measured during and after injection, and abnormalities in laboratory values were recorded.

Seventy patients per group were required to be 80% sure that the lower limit of a 90% two sided confidence interval will exclude a difference in favor of the standard 3 day regimen of more than 15% (non-inferiority margin), assuming a 85% clinical cure for both groups.[11] The 15% non-inferiority margin was determined by consensus of a panel of dermatologists experienced in the treatment of CL and is in line with a similar study.[12]

Differences in proportions were presented with the 90% Confidence Interval (CI). The 3 day regimen was considered non-inferior if the left-side of the 90% CI did not exceed the non-inferior margin of minus 15% except for the difference in proportions of adverse and toxicological events were the right-side of the 90% CI should not exceed the 15%.

Data were analyzed as intention to treat (ITT) and per protocol (PP). For ITT analysis, subjects withdrawn from the study, lost to follow-up, or not attending the evaluation visit, were either considered therapeutic failures or, having an adverse event or abnormal clinical laboratory value and they were excluded from the PP analysis. Furthermore, patients not receiving additional treatment at follow up visit 6 according to protocol were excluded from the PP analyses at follow up visit 12 weeks. The safety data set included individuals who had received at least one injection. To estimate if disease-specific HRLQ scores changed before and six weeks after intervention and to see if the change in HRLQ over time was different between the two groups, a generalized estimation equation with independent correlation structure and a robust estimation of the standard errors were conducted. Age and gender were included in this model. The data set included only those individuals without missing data. HRLQ between groups was compared using the Mann Whitney test and χ^2^ tests were appropriate.

Except for HRLQ, we conducted sensitivity analyses based on the ITT population in which it was assumed that individuals without data on the outcome of interest were considered to be either cured (for clinical and parasitological cure) or not having any adverse events Statistical analyses were performed using STATA SE12.1.

## Results

### General characteristics of study population

**Fig 1:**
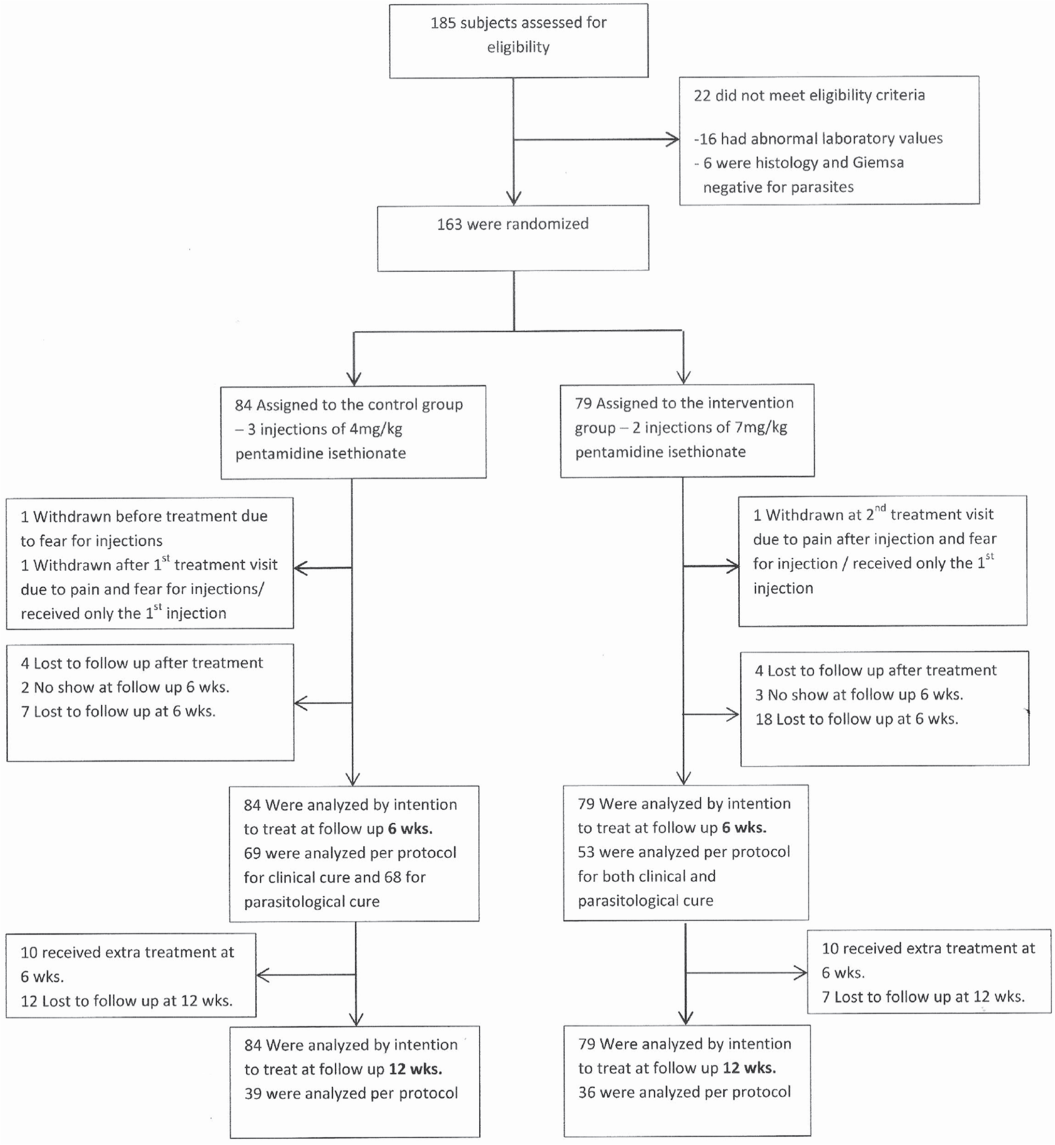
PELESU study in patients with cutaneous leishmaniasis in Suriname from 2010–2013: study enrollment, randomization and follow-up.

From January 3^rd^ 2010 to April 30^th^ 2013, 185 patients with suspected CL were assessed for eligibility. A total of 163 eligible patients were randomized (Fig 1). Study compliance was lower in the 3 day regimen group: at 6 weeks follow-up 32.9% (26/79) was either lost to follow-up (23) or did not show up (3) compared to 17.9% (15/84) in the 7 day regimen group, the difference was statistically significant (p = 0.013). At follow-up visit 12 weeks respectively 40.5% (32/79) and 29.8% (25/84) were lost to follow-up, the difference was not statistically significant (p = 0.074).

There were no differences between the two groups regarding sex, age, ethnicity, occupation, number or location of lesions and presence of regional lymphangitis. (Table 1).

**Table 1:** Baseline characteristics of included patients with cutaneous leishmaniasis in Suriname from 2010–2013 according to treatment group.

Characteristics	7 day regimen (N = 84)	3 day regimen (N = 79)
**Sex**, n (%) Male	77 (91.7%)	73 (92.4%)
**Age** Median (range) year	33 (16–59)	30 (18–75)
**Ethnicity**, n (%)		
Maroon	31 (36.9%)	38 (48.1%)
Amerindian	5 (6.0%)	4 (5.1%)
Creole	5 (6.0%)	6 (7.6%)
Hindustani	21 (25.0%)	10 (12.7%)
Javanese	12 (14.3%)	11 (13.9%)
Others	10 (11.9%0	10 (12.7%)
**Occupation**, n (%)		
Golddigger	13 (15.5%)	18 (22.8%)
Woodchopper	8 (9.5%)	8 (10.1%)
Teacher	0 (0.0%)	1 (1.3%)
Military	3 (3.6%)	4 (5.1%)
Others	60 (71.4%)	48 (60.8%)
**No. of lesions per subject (%)**		
1	44 (52.4%)	40 (50.6%)
2	15 (17.9%)	14 (17.7%)
≥3	25 (29.8%)	25 (31.7%)
**Median no. of lesions (range)**	1 (1–101)	1 (1–81)
**Topography of lesions**, n (%)		
Upper extremities	38 (45.2%)	39 (49.4%)
Lower extremities	48 (57.1%)	40 (50.6%)
Trunk	21 (25.0%)	21 (26.6%)
Head and face	9 (10.7%)	14 (17.7%)
**Presence of regional lymphangitis related to the largest lesion**, n (%)	65 (77.4%)	63 (79.7%)

*Ethnicity: Maroon-descendent of runaway slaves, traditionally living in the interior / Amerindians-indigenous population / Creole-descendants from slaves, traditionally living in urbanized areas / Hindustani-descendants of British-Indian immigrants / Javanese-descendants of Dutch-Indian immigrants.

### Primary endpoint: Clinical cure at 6 weeks

The proportion clinically cured was 39.2% in the 3 day regimen and 48.9% in the 7 day regimen group. ITT analysis showed that the difference in proportion of clinical cure was minus 9.6% (90% CI: -22.3% to 3.2%) (Table 2) and in the PP analysis the difference was 0.2% (90% CI: -14.6% to 15.2%). Because the ITT analyses showed that the left side of the 90% CI exceeded the non-inferior margin of 15% and the results of both types of analyses were not consistent we cannot conclude that the 3 day regimen is non-inferior to the 7 day regimen.

**Table 2:** Efficacy analyses: Responses to treatment at follow-up 6 weeks and 12 weeks after treatment according to treatment of cutaneous leishmaniasis patients in Suriname from 2010–2013.

	7-day regimen	3-day regimen	
**Primary outcome**	N = 84	N = 79	
Did not receive (completed) allocated intervention	2 (2.4%)	1 (1.3%)	
***Clinical cure*: *treatment outcome week 6***			
Cured	41 (48.9%)	31 (39.2%)	
Treatment failure	28 (33.3%)	22 (27.8%)	
Lost to follow-up	13 (15.5%)	23 (29.1%)	
Did not attend visit wk6	2 (2.4%)	3 (3.8%)	
Cured: Intention-to-treat-population ^1^	-9.6 (90% confidence interval: -22.3–3.2) *		
	**N** = 69		**N** = 53
Cured: Per-protocol-population ^1^	0.2 (90% confidence interval: -14.6–15.2)		
**Secondary outcomes**			
**Parasitological cure: treatment outcome week 6**			
Cured	50 (59.5%)	35 (44.3%)	
Treatment failure	18 (21.4%)	18 (22.8%)	
Lost to follow-up	13 (15.5%)	23 (29.1%)	
Did not attend week 6	2 (2.4%)	3 (3.8%)	
No sample	1 (1.2%)	0 (0%)	
Cured: Intention-to-treat-population^1^	-15.2 (90% confidence interval: -28.0 – -2.5) **		
	**N** = 68		**N** = 53
Cured: Per-protocol-population^1^	-6.4 (90% confidence interval: -20.3–7.5) *		
**Clinical cure: treatment outcome week 12**			
Cured	45 (53.6%)	37 (46.8%)	
Treatment failure	14 (16.7%)	12 (15.2%)	
Lost to follow-up	25 (29.8%)	30 (38.0%)	
Cured: Intention-to- treat-population^1^	-6.7 (90% confidence interval: -19.6–6.1) *		
	**n** ^#^ ^=^ 39		**n** ^#^ ^=^ 36
Cured: Per-protocol-population^1^	-6.4 (90% confidence interval-19.4–6.6)		
**Parasitological cure: treatment outcome week 12**			
Cured	46 (54.8%)	29 (36.7%)	
Treatment failure	13 (15.5%)	18 (22.8%)	
Lost to follow-up	25 (29.8%)	30 (38.0%)	
No sample	0 (0%)	2 (2.5%)	
*Successful treatment (cured)*			
Cured: Intention-treat-population^1^	-18.1 (90% CI: -30.7–-5.4) **		
	**n** ^#^ = 39		**n** ^#^ = 36
Cured: Per-protocol-population^1^	-20.7 (90% CI: -37.0 – -4.5) **		

^1^ Proportion of individuals cured in 3-day regimen group minus proportion of individuals cured in 7-day regimen group.

* The left side of the 90% confidence interval exceeds the non-inferior margin of 15%. It can therefore not be concluded that the 3-day regimen is non-inferior to the 7-day regimen.

** The left side of the 90% confidence interval exceeds the non-inferior margin of 15%; furthermore the right-side of the 90% confidence interval and the right side of the 95% confidence interval (not presented) is below 0, indicating that the 3-day regimen is not only non-inferior but the proportion of parasitological cured in this group is also lower than in the 7-day regimen group.

^#^ **7-day regimen**: 20 of 28 patients not clinical cured at follow up-visit week 6 did not accept the additional 3 injections of 300 mg PI as required according to the protocol; 2 of 8 patients not clinical cured but receiving additional injections received less than 3 injections; 1 patient clinical cured according to dermatologists based on clinical pictures was not considered cured by the care provider and received additional 3 injections, 1 person was lost to follow up but did receive additional treatment; overall 4 of these 24 did not had outcome data at follow up week visit 12. In conclusion: 20 patients in the 7 day regimen were not included in the per protocol analyses because of failure with respect to the additional treatment. 3 day regimen: 12 of 22 patients not clinical cured at follow up-visit week 6 did not accept the additional 3 injections of 300 mg PI as required according to protocol; 3 of 10 patients not clinical cured but receiving additional regimen received less than 3 injections; overall 2 of these 15 did not had outcome data at follow up week visit 12. In conclusion: 13 patients in the 3 day regimen were not included in the per protocol analyses because of failure with respect to the additional treatment.

### Secondary endpoints

#### Parasitological cure at 6 weeks

The proportion parasitological cured was 44.3% in the 3 day regimen compared to 59.5% in the 7 day regimen; a difference of minus 15.2% (90% CI:-28.0% to -2.5%) in the ITT analysis and minus 6.4% (90% CI: -20.3% to 7.5%) in the PP analysis. For the ITT population, not only the left side of the 90% CI exceeded the non-inferior margin, also the right side of the 90% CI was lower than 0% (-2.5%) and even the right side of the 95% CI was slightly less than 0% (-0.01%) indicating that the 3 day regimen was not only non-inferior but could even be worse compared to the 7 day regimen.

#### Clinical and parasitological cure at 12 weeks

For both clinical and parasitological cure results at 12 weeks were similar to the 6 weeks treatment outcome. Furthermore, with respect to the difference in proportion parasitological cured the 3 day regimen was not only non-inferior but also worse compared to the 7 day regimen. (Table 2)

#### Health related quality of life (HRQL)

The 2 groups did not differ on any of the SKINDEX-29 domains before injections and at 6 weeks follow-up (Table 3). Before treatment half of the patients experienced at most mildly impaired HRLQ.[13] The SKINDEX-29 score decreased significantly towards the 6 weeks follow-up (β varying from -0.07 to -0.17; p values 0.00); while this decrease did not differ over time between the two groups (p values > 0.05). Increasing age was significantly related with a decreasing score on the dimensions emotions (β -0.17, p 0.003), function (β -0.13, p 0.02) and overall score (β -0.13, p 0.01), indicating that with each increasing year quality of life HRLQ score slightly decreased with 0.13 to 0.17 points. Females had a score 10 to 12 points higher on the dimensions emotions (β 10.1, p value 0.04), functions (β 12.4, p value 0.03) and overall (β 10.2, p value 0.03) compared to males.

**Table 3:** Results of SKINDEX-29 and EQ-5D questionnaires on treatment visit 1 and follow-up visits 6 weeks according to treatment group of cutaneous leishmaniasis patients in Suriname from 2010–2013.

	Before 1^st^ injection		P value#	6 weeks follow up		P value^#^
	7 day regimen median (p25-p75)	3 day regimen median (p25-p75)		7 day regimen median (p25-p75)	3 day regimen median (p25-p75)	
**Skindex-29**						
*N of individuals* *	83	79		69	53	
Symptoms	39.3 (28.6–42.9)	39.3 (35.7–42.9)	0.14	0.0 (0.0–10.7)	0.0 (0.0–7.1)	0.15
Emotions	22.5 (17.5–30.0)	25.0 (20.0–40.0)	0.14	2.5 (0.0–10.0)	0.0 (0.0–10.0)	0.99
Functions	27.1 (0.0–97.9)	29.2 (18.8–39.6)	0.18	0.0 (0.0–4.2)	0.0 (0.0–2.1)	0.82
Overall	28.4 (22.4–35.3)	31.0 (24.1–38.8)	0.11	1.7 (0.0–7.6)	1.7 (0.0–6.0)	0.6
**EQ 5D**	**7 day regimen n (%)**	**3 day regimen n (%)**		**7 day regimen n (%)**	**3 day regimen n (%)**	
*N of individuals* *	83 (100%)	79 (100%)		69 (100%)	53 (100%)	
Mobility	16 (19.3%)	12 (15.2%)	0.49	2 (2.9%)	0 (0%)	0.21
Self-care	36 (43.4%)	31 (39.2%)	0.59	2 (2.9%)	1 (1.9%)	0.72
Usual activities	49 (59.0%)	36 (45.6%)	0.09	5 (7.2%)	1 (1.9%)	0.18
Pain/discomfort	44 (53.0%)	44 (55.7%)	0.73	5 (7.2%)	3 (5.7%)	0.73
Anxiety/depression	42 (50.6%)	40 (50.6%)	0.99	2 (2.9%)	5 (9.4%)	0.12
**EQ-VAS**						
Median (p25-p75)	81 (75–90)	80 (75–90)	0.47	93 (90–97)	90 (90–95)	0.01

* One patient in the 7 day regimen withdrew before the 1^st^ treatment visit; 15 patients in the 7 day regimen group and 26

patients in the 3 day regimen group did not show up.

# P value: Mann Whitney test for continuous outcomes

Chi^2^ test for categorical outcomes.

#### Safety analyses

In general PI as study medication was well tolerated. No serious toxicity occurred and none of the reported side effects required discontinuation of treatment in any patient. Pain at the injection site was seen most frequently, 77.1% of patients in the 7 day regimen and 81.0% in the 3 day regimen (Table 4). Non-inferiority of the 3 day regimen over the 7 day regimen based on side effects could not be proved for the majority of the side effects while taste change occurred even more frequently in the 3 day regimen. PP analyses showed similar results.

**Table 4:** Safety analyses: Adverse events 1 week after treatment according to treatment group of cutaneous leishmaniasis patients in Suriname from 2010–2013 #.

Side effects		7 day regimen (N^1^ = 84)	3 day regimen (N^1^ = 79)	Difference in proportion (90% Confidence Interval) ITT analyses^#^	Difference in proportion (90% Confidence Interval) PP analyses^$^
**Received at least 1 injection (N** ^**2**^)		83	79		
**Valid observations (N** ^**3**^)		78	74		
**Missing values**		5	5		
**Nausea**	Yes	20 (24.1%)	24 (30.4%)	6.6 (-5.6–18.8)*	6.8 (-5.3–18.9) *
	No	58 (69.9%)	50 (63.3%)		
**Vomiting**	Yes	1 (1.2%)	4 (5.1%)	4.2 (-3.0–11.3)	4.1 (-0.6–8.9)
	No	77 (92.8%)	70 (88.6%)		
**Fever**	Yes	19 (22.9%)	27 (34.2%)	11.6 (-0.6–23.8)*	12.1 (-0.1–24.3)*
	No	59 (71.1%)	47 (59.5%)		
**Skin rash**	Yes	3 (3.6%)	5 (6.3%)	3.0 (-5.1–11.1)	2.9 (-3.1–8.9)
	No	75 (90.4%)	69 (87.3%)		
**Abdominal pain**	Yes	14 (16.9%)	17 (21.5%)	5.0 (-6.3–16.2)*	5.0 (-5.7–15.8)*
	No	64 (77.1%)	57 (72.2%)		
**Taste change**	Yes	39 (47.0%)	59 (74.7%)	28.0 (16.4–39.6)*	29.7 (17.7–41.8)*
	No	39 (47.0%)	15 (19.0%)		
**Dizziness**	Yes	19 (22.9%)	17 (21.5%)	-1.1 (-12.7–10.6)	-1.4 (-12.7–10.0)
	No	59 (71.1%)	57 (72.2%)		
**Pain at injection site**	Yes	64 (77.1%)	64 (81.0%)	4.2 (-4.9–13.4)	-4.4 (-5.3–14.1)
	No	14 (16.9%)	10 (12.7%)		
**Swelling at injection site**	Yes	40 (48.2%)	47 (59.5%)	11.6 (-1.0–24.2)*	12.2 (-0.9–25.3)*
	No	38 (45.8%)	27 (34.2%)		
**Infection at injection site**	Yes	0 (0.0%)	0 (0.0%)	0.0 (-5.9–6.5)	0.0 (0.0)
	No	78 (94.0%)	74 (93.7%)		
**Other side effects**	Yes	9 (10.8%)	17 (21.5%)	11.0 (0.3–21.7)*	11.4 (1.4–21.4)*
	No	69 (83.1%)	57 (72.2%)		

^1^ Number of individuals randomized; N^2^ Number of individuals who received at least one injection, the denominator for the safety analyses; N^3^ Number of individuals without missing data one week after receiving the last injection.

# Adverse events were evaluated in all patients who received at least one injection. Individuals with missing data (5 in each group) were considered to have the outcome of interest.

$ Individuals with missing were excluded from analyses.

* The right side of the 90% confidence interval is higher than the non-inferior margin of 15% indicating that the 3 day intervention is not non-inferior compared to the 7 day intervention.

For hemoglobin, creatinine and amylase levels we could not prove non-inferiority of the 3 day regimen over the 7 day regimen based on laboratory findings. Creatine levels were even significantly higher in the 3 day group (Table 5). In the PP analyses absence of non-inferiority was only showed for pancreas toxicity.

**Table 5:** Clinical laboratory values at follow-up 1 week after last treatment according to treatment group of cutaneous leishmaniasis patients in Suriname from 2010–2013#.

Laboratory values		7 day regimen N^1^ = 83	3 day regimen N^1^ = 79	Difference in proportion (90% Confidence Interval) ITT analyses^#^	Difference in proportion (90% Confidence Interval) PP analyses^$^
Received at least 1 injection (N^2)^		83	79		
**Hemolysis (<7.5 mmol/l)**	Yes	1 (1.2%)	5 (6.3%)	8.0 (-1.7–16.1)*	5.7 (0.3–11.0)
	No	77 (92.8%)	67 (84.8%)		
	Missing values	5 (6.0%)	7 (8.9%)		
**Leucopenia (<4x10** ^**9**^/**l**)	Yes	9 (10.8%)	7 (8.9%)	0.9 (-8.9–10.6)	-1.8 (-10.1–6.5)
	No	69 (83.1%)	65 (82.3%)		
	Missing value	5 (6.0%)	7 (8.9%)		
**Thrombocytopenia (<150x10** ^**9**^/**l**)	Yes	0 (0.0%)	0 (0.0%)	2.8 (-4.0–9.6)	0 (0–0)
	No	78 (94.0%)	72 (91.1%)		
	Missing values	5 (6.0%)	7 (8.9%)		
**Hypoglycemia (glucose <4mmol/l)**	Yes	6 (7.3%)	2 (2.6%)	-3.1 (-11.4–5.2)	-5.0 (-10.8–0.9)
	No	72 (87.8%)	71 (91.0%)		
	Missing values	4 (4.9%)	5 (6.4%)		
**Hyperglycemia (glucose >6.5mmol/l)**	Yes	4 (4.8%)	6 (7.6%)	4.3 (-4.4–13.0)	3.1 (-3.6–9.8)
	No	74 (89.2%)	67 (84.8%)		
	Missing values	5 (6.0%)	6 (7.6%)		
**Nephrotoxicity (kreatine >120umol/l**	Yes	0 (0%)	6 (7.7%)	9.2 (1.3–17.1)*	8.2 (2.9–13.5)
	No	78 (95.1%)	67 (85.9%)		
	Missing values	4 (4.9%)	5 (6.4%)		
**Pancreas toxicity (amylase >96umol/l)**	Yes	5 (6.0%)	11 (13.9%)	9.5 (-0.1–19.1)*	8.7 (0.3–16.7)*
	No	73 (88.0%)	62 (78.5%)		
	Missing values	5 (6.0%)	6 (7.6%)		
**Liver toxicity (SGPT >80umol/l)**	Yes	4 (4.8%)	4 (5.1%)	2.0 (-7.4–11.3)	0.4 (-5.9–6.6)
	No	71 (85.5%)	66 (83.5%)		
	Missing values	8 (9.6%)	9 (11.4%)		
**Liver toxicity (SGOT >80umol/l)**	Yes	1 (1.2%)	3 (3.8%)	4.3 (-4.0–12.6)	2.9 (-1.6–7.4)
	No	75 (90.4%)	68 (86.1%)		
	Missing values	7 (8.4%)	8 (10.1%)		

N^1^ Number of individuals randomized; N^2^ Number of individuals who received at least one injection, the denominator for the safety analyses;

# Clinical laboratory values were evaluated in all patients who received at least one injection at one week after receiving the last injection. Individuals with missing data were considered to have the outcome of interest for the ITT analyses;

$ Individuals with missing data (including two individuals who only received one injection and were lost to follow up) were excluded from PP analyses;

* The right side of the 90% confidence interval exceeds the non-inferior margin of 15% indicating that with respect to these clinical laboratory values it cannot be concluded that the 3 day regimen is non-inferior compared to the 7 day regimen.

During the first injection, a drop in the diastolic BP of > 10 mmHg was recorded in 2.5% (2/79) in the 3 day regimen patients and 2.4% (2/83) in the 7 day regimen patients: difference 0.1% (90% CI: -3.9% to 4.1%). Thirty minutes after the 1^st^ injection 8.9% (90% CI: -3.0%–14.8%) more patients in the 3 day regimen had a drop in the diastolic BP > 10mmHG, 10.1% (8/79) compared to 1.2% (1/83) in the 7 day regimen.

#### Sensitivity analyses

Sensitivity analyses comparing missing data as successes showed that the 3 day regimen was non-inferior to the 7 day regimen: 5.5% (90% CI: -6.4% to 17.3%) and minus 1.4% (90% CI: -12.1% to 9.3%) at 6 weeks outcome for clinical and parasitological cure respectively. Twelve weeks after end of treatment non-inferiority was shown for clinical cure (1.5%; 90% CI: -7.9% to 10.9%) but not for parasitological cure (-13.8; 90% CI: -30.8% to 3.3%).

Sensitivity analyses of the safety data set revealed that for adverse events the results were similarly not non-inferior to the ITT and PP analyses (S1 and S2 Tables).

## Discussion

We conducted a non-inferiority trial because we intended to show that the new 3 day regimen was not worse compared to the standard 7 day regimen. Our results showed that the 3 day regimen is not non inferior to the standard treatment (7 days, three IM injections PI of 4 mg/kg) regarding clinical cure at 6 weeks. Therefore, we cannot recommend the 3 day regimen over the 7 day regimen to treat CL in Suriname. Although the 90% CI for the ITT analyses are slightly lower than the non-inferiority margin of 15%, consistency between PP and ITT analyses is an important requisition in non-inferiority trials before conclusions regarding non-inferiority can be drawn.[14,15] The other clinical and parasitological secondary endpoints support the main finding.

Nonetheless sensitivity analyses (based on the assumption that all lost to follow patients were cured) showed non-inferiority, except for parasitological cure at 12 weeks. Seven lost to follow-up patients in the 7 day regimen and six in the 3 day regimen could be reached by telephone. All confirmed that their lesions had healed. However, we also know from clinical practice that non-cured patients sometimes sought help in traditional medicine.[16]

Based on an average found in previous studies in Suriname we assumed a cure rate of 85% for both groups.[3,4] In the PP analysis the proportion clinical cured at 6 weeks follow-up was 58% in the 3 day regimen and 59% in the 7 day regimen and 76% in both groups at 12 weeks. The lower cure rate at week 6 in our study may either be due to the early evaluation moment at which not all lesions had healed, but might also be a consequence of the high percentage lost to follow-up with unknown outcome.

The safety analyses should be considered as explorative data analyses because the power of the study was based on the primary endpoint and the issue of multiplicity arising from numerous comparisons may arise. The latter may explain why taste change occurred more frequently in the 3 day regimen while for the remaining safety analyses no differences were found. The absence of non-inferiority for several adverse events and laboratory values may be due to the higher concentration of PI per injection in the 3 versus the 7 day regimen.

One of the limitations of this study is that we cannot mention the causative species affecting our participants. Since the far majority of CL in Suriname is caused by L. *guyanensis* and other spiecies like L. *amazonensis*, L. *braziliensis* and L. *naffï* have only been reported sporadically,[17,18,19] we considered all patients to be infected by L. *guyanensis*.

Two studies using the Dermatology Quality of Life Scale found lower HRQL in patients with active CL compared with healed CL patients.[20, 21] We are the first to assess the HRQL in CL using the Skindex-29 and EQ-5D/VAS. Our results showed improvement in HRQL in both treatment groups at 6 weeks follow up visit. The dimensions emotions, functioning and the overall score of Skindex-29 were positively associated with older age. Gurel MS et al. also showed that older people had fewer problems with CL lesions and scars after healing while youngsters accepted lesions and scar formation after healing less well.[21] Females had higher scores in the dimensions emotions, functioning and overall in the Skindex-29 compared to males. In Pakistan and Afghanistan female CL patients also reported higher HRQL parameters than males.[22]

A priori we hypothesized that the 3 day regimen would have resulted in a decreased number of individuals lost to follow up for the control visits. However, in the 3 day regimen the percentage lost to follow-up at 6 weeks was even higher with 33% versus 18% in the 7 day regimen. Although not statistically significant, this could be attributed to more side effects in the 3 day regimen. Another explanation could be a higher portion of healed patients in the 3 day regimen without a need for return visits. Unfortunately we were unable to conduct active follow-up as most patients returned to the hinterlands and could not be contacted. The high number of lost to follow-up may have biased the study results, especially because the percentage of individual’s lost to follow-up where higher in the 3 day regimen. Because the sensitivity analyses showed non inferiority of the 3 day regimen there is indeed indication that the high percentage of loss to follow-up has biased the study results. Furthermore, it is possible that the power of our study was too low to detect non-inferiority because the clinical and parasitological cure rates were lower than assumed in the power calculation (85%). Too low power in a non-inferiority trial indicates that there is more chance to falsely conclude that the intervention is not non-inferior while the intervention is non-inferior. A retrospective study performed in Suriname in 2001 in which patients were treated with pentamidine from 1979 till 2000 found a cure rate of 90% after a follow-up of 3–6 months.[3] Since that time resistance could have cause a decline in the cure rate. Moreover assessment of the cure rate after 6 weeks instead of 3–6 months can explain the lower cure rate found in our study.

Since we knew from standard clinical practice that compliance is low in CL patients, those who doubted if they could attend all follow-up visits were not eligible. As a result, our study may suffer from a lower external validity because the study population might not be completely representative for the CL patient population in Suriname.

In future studies serious attention should be given to reduce the number of lost to follow-up patients, for example via financial compensation for study related expenses. Moreover, novel oral CL treatment like miltefosine instead of painful PI injections could improve compliance.[23]

In conclusion, we cannot conclude that two injections of 7 mg/kg PI within three days is non-inferior to the standard regimen of three injections of 4 mg/kg PI within 7 days, for treatment of CL in Suriname. Therefore, there is no evidence to change the current standard practice of the 7 day regimen treatment for CL in Suriname.

## Supporting Information

S1 Checklist Consort checklist. Randomized single-blinded non-inferiority trial of 7 mg/kg pentamidine isethionate versus 4 mg/kg pentamidine isethionate for cutaneous leishmaniaisis in Suriname
(PDF)

S1 Table Sensitivity analyses of Safety data. Adverse events 1 week after treatment according to treatment group of cutaneous leishmaniasis patients in Suriname from 2010–2013.
(DOCX)

S2 Table Sensitivity analyses of clinical laboratory values at follow-up 1 week after last treatment according to treatment group of cutaneous leishmaniasis patients in Suriname from 2010–2013. (DOCX)
