# Supplementary material for: Randomized Single-Blinded Non-inferiority Trial Of 7 mg/kg Pentamidine Isethionate Versus 4 mg/kg Pentamidine Isethionate for Cutaneous Leishmaniaisis in Suriname
Source: PLoS Negl Trop Dis. 2015 Mar 20;9(3):e0003592. doi: 10.1371/journal.pntd.0003592 (PMC4368628; doi:10.1371/journal.pntd.0003592)
Supplement: S1 Table — Adverse events 1 week after treatment according to treatment group of cutaneous leishmaniasis patients in Suriname from 2010–2013. (DOCX) [file pntd.0003592.s002.docx]

| **Side effects** | | **7 day regimen**  **(N^1^=84)** | **3 day regimen**  **(N^1^=79)** | **Difference in proportion**  **(90% Confidence Interval)**  **Sensitivity analyses** |
| --- | --- | --- | --- | --- |
| **Received at least 1 injection (N^2^)** | | 83 | 79 |  |
| **Missing values ^#^** | | 5 (6.0%) | 5 (6.3%) |  |
| **Nausea** | Yes  No | 20 (24.1%)  63 (75.9%) | 24 (30.4%)  55 (69.6%) | 6.6 (-5.2-17.8) * |
| **Vomiting** | Yes  No | 1 (1.2%)  82 (98.8%) | 4 (5.1%)  75 (94.9%) | 3.9 (-.7-8.4) |
| **Fever** | Yes  No | 19 (22.9%)  64 (77.1%) | 27 (34.2%)  52 (65.8) | 11.3 (-0.3-22.9) * |
| **Skin rash** | Yes  No | 3 (3.6%)  80 (96.4) | 5 (6.3%)  74 (94.7%) | 2.7 (-2.9-8.3) |
| **Abdominal pain** | Yes  No | 14 (16.9%)  69 (83.1) | 17 (21.5%)  62 (78.5) | 4.7 (-5.5-14.8) |
| **Taste change** | Yes  No | 39 (47.0%)  44 (53.0%) | 59 (74.7%)  20 (25.3%) | 27.7 (15.6-39.8) * |
| **Dizziness** | Yes  No | 19 (22.9%)  64 (77.1%) | 17 (21.5%)  62 (78.5) | -1.4 (-12.1-9.4) |
| **Pain at injection**  **site** | Yes  No | 64 (77.1%)  19 (22.9%) | 64 (81.0%)  15 (19.0%) | 3.9 (-6.6-14.4) |
| **Swelling at**  **injection site** | Yes  No | 40 (48.2%)  43 (51.8%) | 47 (59.5%)  32 (40.5%) | 11.3 (-1.5-24.1) * |
| **Infection at**  **injection site** | Yes  No | 0 (0%)  83 (100.%) | 0 (0%)  79 (100%) | - |
| **Other side effects** | Yes  No | 9 (10.8%)  74 (89.2%) | 17 (21.5%)  62 (78.5%) | 10.7 (1.2-20.1) * |

N^1^ Number of individuals randomized; N^2^ Number of individuals who received at least one injection, the denominator for the safety analyses; # Individuals with missing data (5 in each group) were considered to not having adverse events one week after receiving the last injection; * The right side of the 90% confidence interval is higher than the non-inferior margin of 15% indicating that the 3 day intervention is not non-inferior compared to the 7 day intervention.
