# Supplementary material for: Randomized Single-Blinded Non-inferiority Trial Of 7 mg/kg Pentamidine Isethionate Versus 4 mg/kg Pentamidine Isethionate for Cutaneous Leishmaniaisis in Suriname
Source: PLoS Negl Trop Dis. 2015 Mar 20;9(3):e0003592. doi: 10.1371/journal.pntd.0003592 (PMC4368628; doi:10.1371/journal.pntd.0003592)
Supplement: S2 Table — (DOCX) [file pntd.0003592.s003.docx]

| **Laboratory values** | | **7 day regimen**  **N^1^ =83** | **3 day regimen**  **N^1^ =79** | **Difference in proportion**  **(90% Confidence Interval)** |
| --- | --- | --- | --- | --- |
| **Received at least 1 injection (N^2^)** | | **83** | **79** |  |
| **Hemolysis**  **(<7.5 mmol/l)** | Yes  No | 1 (1.2%)  82 (98.8%) | 5 (6.3%)  74 (93.7%) | 5.0 (0.2-10.0) |
| **Leucopenia**  **(<4 x10^9^ /l)** | Yes  No | 9 (10.8%)  74 (89.2%) | 7 (8.9%)  72 (91.1%) | -1.9 (-9.7-5.7) |
| **Thrombocytopenia**  **(<150 x10^9^ /l)** | Yes  No | 0 (0%)  83 (100.0%) | 0 (0%)  79 (100%) | - |
| **Hypoglycemia**  **(glucose< 4mmol/l)** | Yes  No | 6 (7.3%)  76 (92.7%) | 2 (2.6%)  76 (97.4%) | -4.7 (-10.0-0.8) |
| **Hyperglycemia**  **(glucose> 6.5mmol/l)** | Yes  No | 4 (4.8%)  79 (95.2%) | 6 (7.6%)  73 (92.4%) | 2.8 (-3.5-9.0) |
| **Nephrotoxicity**  **(kreatine> 120umol/l)** | Yes  No | 0 (0%)  82 (100%) | 6 (7.7%)  72 (92.3%) | 7.6 (2.7-12.5) |
| **Pancreas toxicity**  **(amylase> 96umol/l)** | Yes  No | 5 (6.0%)  78 (94.0%) | 11 (13.9%)  68 (68.1%) | 7.9 (0.2-15.6)* |
| **Liver toxicity**  **-SGPT >80umol/l**  **-SGOT >80umol/l** | Yes  No  Yes  No | 4 (4.8%)  79 (95.2%)  1 (1.2%)  82 (98.8%) | 4 (5.1%)  75 (94.9%)  3 (3.8%)  76 (96.2%) | 0.2 (-5.4-5.8)  2.6 (-1.5-6.6) |

N^1^ Number of individuals randomized; N^2^ Number of individuals who received at least one injection, the denominator for the safety analyses; # Clinical laboratory values were evaluated in all patients who received at least one injection at one week after receiving the last injection. Individuals with missing data were considered to have no adverse events. The right side of the 90% confidence interval exceeds the non-inferior margin of 15% indicating that with respect to these clinical laboratory values it cannot be concluded that the 3 day regimen is non-inferior compared to the 7 day regimen.
